# A directed acyclic graph for interactions

**DOI:** 10.1093/ije/dyaa211

**Published:** 2020-11-22

**Authors:** Anton Nilsson, Carl Bonander, Ulf Strömberg, Jonas Björk

**Affiliations:** 1 EPI@LUND (Epidemiology, Population Studies and Infrastructures at Lund University), Lund University, Lund, Sweden; 2 Centre for Economic Demography, Lund University, Lund, Sweden; 3 School of Public Health and Community Medicine, Sahlgrenska Academy, University of Gothenburg, Gothenburg, Sweden; 4 Department of Research and Development, Region Halland, Halmstad, Sweden; 5 Clinical Studies Sweden, Forum South, Skåne University Hospital, Lund, Sweden

**Keywords:** Causal inference, external validity, generalizability, interaction, internal validity, mediation

## Abstract

**Background:**

Directed acyclic graphs (DAGs) are of great help when researchers try to understand the nature of causal relationships and the consequences of conditioning on different variables. One fundamental feature of causal relations that has not been incorporated into the standard DAG framework is interaction, i.e. when the effect of one variable (on a chosen scale) depends on the value that another variable is set to. In this paper, we propose a new type of DAG—the interaction DAG (IDAG), which can be used to understand this phenomenon.

**Methods:**

The IDAG works like any DAG but instead of including a node for the outcome, it includes a node for a causal effect. We introduce concepts such as confounded interaction and total, direct and indirect interaction, showing that these can be depicted in ways analogous to how similar concepts are depicted in standard DAGs. This also allows for conclusions on which treatment interactions to account for empirically. Moreover, since generalizability can be compromised in the presence of underlying interactions, the framework can be used to illustrate threats to generalizability and to identify variables to account for in order to make results valid for the target population.

**Conclusions:**

The IDAG allows for a both intuitive and stringent way of illustrating interactions. It helps to distinguish between causal and non-causal mechanisms behind effect variation. Conclusions about how to empirically estimate interactions can be drawn—as well as conclusions about how to achieve generalizability in contexts where interest lies in estimating an overall effect.

**Key Messages**

- Directed acyclic graphs (DAGs) are useful in epidemiology, but the standard framework offers no way of displaying whether interactions are present (on the scale of interest).
- We present a new type of DAG—the interaction DAG (IDAG)—which can be used to analyse interactions.
- We define concepts such as confounded interaction and total, direct and indirect interaction, and show how these can easily be displayed with the IDAG.
- An applied researcher can use the IDAG to determine which treatment interactions to account for empirically.
- The IDAG can also be used to shed light on mechanisms that compromise generalizability and to determine which variables to account for in order to make results valid for the target population.

## Background

Directed acyclic graphs (DAGs)^1–4^ are frequently used in epidemiology to shed light on causal relationships. Being composed of nodes, representing variables, and arrows, representing direct causal effects of one variable on another, DAGs can be used to illustrate concepts such as confounding, selection bias and the distinction between total, direct, and indirect effects. In turn, DAGs are used to determine which variables to condition on in empirical analyses. Whereas DAGs are powerful tools, a fundamental feature of causal relations which has not been incorporated into the standard framework is interaction, i.e. when the effect of some variable A (on a chosen scale) depends on the value to which another variable Q is set.^5^^,^^6^

Several articles have discussed interaction with reference to DAGs.^7–12^ The standard DAG is nonparametric and as a result, it is of no relevance for the construction of the graph whether the determinants of an outcome interact with each other. There are some proposals on how interaction could intuitively be incorporated into DAGs, but these lack theoretical foundations.^8^

In this article, we propose a new type of DAG, the interaction DAG (IDAG). The IDAG is both intuitive and well founded in theory for causal inference. In brief, the IDAG works like any DAG but instead of depicting how different variables influence the outcome, the IDAG depicts how different variables influence the size of a chosen effect measure. We describe the approach and discuss several concepts that naturally follow from the framework, such as confounded interaction and direct, indirect and total interaction. For readers unfamiliar with standard DAGs, we refer to Greenland,^2^ who provides an accessible introduction.

## The IDAG

The concept of interaction employed in this article is similar to that in previous literature,^5^^,^^6^^,^^10^ and refers to a joint effect. Whereas there are different ways of defining an ‘effect’, the general idea behind interaction is that the effect of one variable (on some scale) depends on the level to which another variable is set. Here, we will focus on a binary treatment A that may interact with one or several other binary variables, such as Q and X.

Definitions of interaction are often expressed with potential outcomes.^13^^,^^14^ In structural causal models, a potential (or ‘counterfactual’) outcome $Y_{i}^{a,q}$ is an outcome that, for a full set of predetermined background factors which characterize individual i, prevails when forcing one or several variables in the model to assume particular values.^15^ When defining interactions, at least two variables must be forced to particular values.

If the outcome Y is continuous, we can say that there is additive interaction between Q and A in individual i if the following inequality holds between differences of potential outcomes:

(1) $$Y_{i}^{a=1,q=1}-Y_{i}^{a=0,q=1}\neq Y_{i}^{a=1,q=0}-Y_{i}^{a=0,q=0}$$

Notice that we here define interaction at the individual level, in some contrast with previous literature, which focuses on the expected population level. The left-hand side of equation (1) is a measure of the causal effect of A on Y for Q=1 , and the right-hand side is the same measure for Q=0. Interaction between Q and A is thus present if the size of this causal effect depends on Q. The size of the interaction is given by the difference between the left-hand and right-hand sides of (1).

When outcomes are binary, focus normally lies on the probability of a positive outcome. Assuming probabilistic potential outcomes,^16^ we can say that additive interaction between Q and A is present in individual i if the following inequality holds in this individual:

(2) $$P[Y_{i}^{a=1,q=1}=1]-P[Y_{i}^{a=0,q=1}=1]\neq P[Y_{i}^{a=1,q=0}=1]-P[Y_{i}^{a=0,q=0}=1]$$

Again, the left-hand side is a measure of the causal effect of A on Y for Q=1 , and the right-hand side for Q=0; the interaction is present if the size of this causal effect depends on Q.

Henceforth, we will denote a causal effect of A on Y by $\Delta Y_{A}$. Since $\Delta Y_{A}$ is a variable that may depend causally on other variables, it can be included in a causal graph. We refer to a graph including $\Delta Y_{A}$ as an IDAG. If there is an interaction between some variable and A, there is a directed arrow (or path) from this variable to $\Delta Y_{A}$. In contrast, effect measure modification only corresponds to an association between some variable and $\Delta Y_{A}$, possibly arising through unblocked backdoor paths. In the Supplementary Appendix, available as Supplementary data at *IJE* online, we discuss more technical details related to the IDAG, such as d-separation,^1^ and work through examples based on structural equations. The IDAG is quite similar to the standard DAG, except that the outcome node has been replaced by a node representing a causal effect, and that the node representing the treatment variable A is not included. Both figures display causal relationships between variables, and the causal effect of one variable on another is not dependent on the graph. Like any DAG, the IDAG will normally be drawn based on previous literature, which in the case of the IDAG will have to include evidence on which treatment interactions are present.

Causal effects can be measured on different scales; for example, although equations (1) and (2) defined interaction on additive scales (based on differences), multiplicative scales (based on ratios) could be used as well. Whether an interaction is present may depend on the scale and, in fact, two variables that influence an outcome will always interact on some scales.^5^^,^^17^^,^^18^ The appearance of the IDAG thus depends on the scale chosen, and certain variables may point to $\Delta Y_{A}$ in some versions of the IDAG but not in others. In general, the additive scale is preferred if the goal is to evaluate interaction in a ‘mechanistic’ sense.^5^^,^^6^^,^^19^

For simplicity, we will assume that there are no interactions not involving A (on the chosen scale), and for this reason we only consider $\Delta Y_{A}$ and not, for example, $\Delta Y_{Q}$. We will also assume that interactions are constant across individuals, so the individual-level interactions defined from equations (1) and (2) are equal to conventional population-level interactions.

## Examples

**Figure 1:**
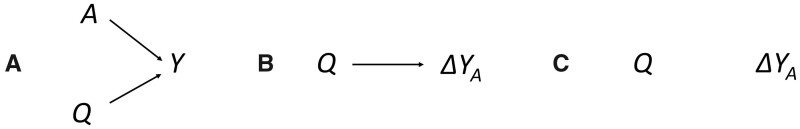
An example of a standard directed acyclic graph (DAG) (panel A) and two possible interaction DAGs (IDAGs) (panels B and C). Variables *A* (warfarin) and *Q* (smoking) influence *Y* (ischaemic stroke). Panel B suggests that *Q* also influences the effect of *A* on *Y*, whereas panel C suggests that this is not the case

We now present several examples of IDAGs, explaining their interpretation and connection to standard DAGs. First, in Figure 1A, we provide a standard DAG. The outcome Y, say ischaemic stroke, is assumed to be influenced by a treatment A and also Q (say, warfarin and smoking), and we want to display whether these two variables interact (say, on an additive scale). Indeed, whether there is such an interaction between the variables is not visible from the standard DAG. This, however, can be seen in the IDAG in Figure 1B, according to which the effects of A are influenced by Q.

The graph in Figure 1B is not the only possible IDAG to accompany the standard DAG in Figure 1A. One could also conceive of an IDAG without an arrow from Q to $\Delta Y_{A}$, i.e. a scenario with no interaction between A and Q. We show this alternative in Figure 1C (in practice, Q could have been omitted from this figure).

As can be noticed, a node Q with an arrow pointing to Y in the standard DAG does not necessarily have an arrow pointing to $\Delta Y_{A}$ in the IDAG. On the other hand, there can be no arrow from Q to $\Delta Y_{A}$ in the IDAG unless Q points to Y in the standard DAG. This follows because the treatment effect depends on the outcomes, so only if a variable directly influences the outcomes may it also directly influence the effect size.

**Figure 2:**
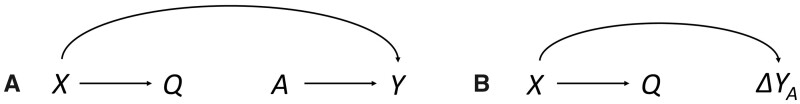
Confounded interaction or ‘effect modification by proxy’. A standard directed acyclic graph (DAG) is given in panel A and an interaction DAG (IDAG) in panel B. Variables *X* (genotype) and *A* (bariatric surgery) influence *Y* (weight loss), with an interaction present. The effect of *A* is modified by *Q* (hair colour), but there is no interaction between *A* and *Q*

Another example of a standard DAG and an accompanying IDAG is given by Figure 2. We consider a scenario where a (perhaps naïve) researcher is asking whether there is an interaction between a treatment, such as bariatric surgery, A, and hair colour, Q, on weight loss Y (on an additive scale). There is an unobserved variable X (genotype) that influences the outcome and that also interacts with treatment—the latter illustrated by an arrow to the causal effect in the IDAG. X also influences hair colour, which does not itself influence the outcome. The relationship between X and Q is indicated in both the standard DAG and the IDAG. Notably, since Q is influenced by X, the effects of A will vary by Q even though there is no interaction between Q and A. The phenomenon has been referred to as ‘effect modification by proxy’^7^ and is an instance of confounded interaction, since a simple analysis of a possible interaction between Q and A will give biased estimates due to the interaction between X and A.

**Figure 3:**
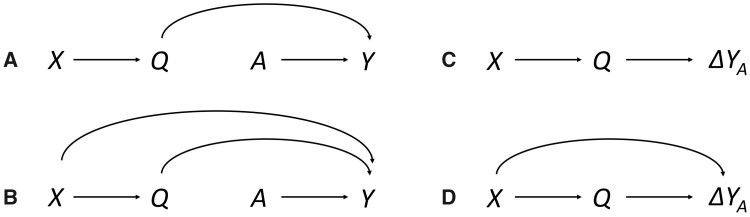
Two examples of standard directed acyclic graphs (DAGs) (left) and two interaction DAGs (IDAGs) (right). The variable *Y* (a disease) is directly influenced by *A* (treatment), *Q* (smoking) and potentially also *X* (education). The DAG in panel A is compatible with the IDAG in panel C, whereas the DAG in panel B is compatible with either of the IDAGs in panels C and D

Further examples of standard DAGs and IDAGs are given in Figure 3, where Q is assumed to influence the outcome. X could represent education and Q smoking; A again is a treatment and Y the disease outcome. We are interested in whether the benefits of treatment (on an additive scale) depend on smoking or education (i.e. interactions between treatment and smoking or education), and whether the potential impact of education on the benefits of treatment are due to the fact that education influences smoking. In Figure 3A, we assume that X has no direct impact on the outcome, whereas such an impact is allowed for in Figure 3B. Figure 3C shows an IDAG compatible with either of the two standard DAGs. Here, it becomes clear that Q and A interact; the arrow from Q to ${\Delta Y}_{A}$ indicates direct interaction. However, there is only indirect interaction with respect to the variable X; once Q is fixed, it makes no difference for the causal effect what value X assumes. Changing educational levels would only influence the benefits of treatment to the extent smoking is influenced.

An alternative IDAG is displayed in Figure 3D. Here, there is direct interaction with respect to both Q and X. As for the effects of X, we can distinguish between direct and total interaction, where the latter operates both directly and indirectly. Increasing educational levels could both influence the benefit of treatment indirectly by reducing smoking, and directly, through other mechanisms omitted from the graph (e.g. adherence). Treatment decisions should here take both the individual’s educational level and smoking status into account, whereas in scenario 3 C it would be enough to take smoking into consideration. Figure 3D is compatible with the DAG in Figure 3B but not with the one in Figure 3A, as in Figure 3A there is no direct impact of X on the outcome.

## Estimation

Typical approaches to estimate an interaction between two variables (Q and A) include stratification and estimation of one regression on the full data, including the product term QA. When used together with the standard DAG, the IDAG provides guidance on how to carry out estimations. Regarding confounding, a sufficient criterion for unconfoundedness in interaction models is that both interacting variables are unconfounded.^5^^,^^10^ For simplicity, our figures have so far ignored the possibility of confounding of the variable A, but in general, variables will need to be conditioned on to make sure A as well as Q is unconfounded. Conclusions about which variables to condition on can be drawn from the standard DAG. However, the standard DAG is uninformative as to what extent stratification or inclusion of product terms is necessary, as opposed to simply controlling for main effects.

To illustrate this point, consider the standard DAG in Figure 3B. In order to estimate the joint effect of Q and A, it is generally necessary to account for X, for example by controlling for it in a regression model, at least including a main term. However, whether it is also necessary to stratify on X or include a product term between X and A depends on whether X influences the causal effect of A on Y (conditional on Q). In the IDAG in Figure 3D, causal effects depend on X, giving rise to a backdoor path between Q and $\Delta Y_{A}$ through X. An analysis examining the interaction between Q and A also needs to account for the interaction between X and A; failure to do so would result in confounded interaction. In the IDAG in Figure 3C, however, causal effects do not depend on X conditional on Q, so it would be enough to control for X with a main term. This is reflected by the absence of a backdoor path between Q and $\Delta Y_{A}$. The reasoning is similar to standard DAG logic; we refer to the Supplementary Appendix, available as Supplementary data at *IJE* online, for more details and elaborations.

Conclusions about what to condition on to estimate total or direct effects follow from both the standard DAG and IDAG. In Figure 3, for example, one must not account for Q (i.e. must omit Q and the interaction between Q and A) to estimate the total effect of X and the total interaction between X and A. In contrast, if interest lies in the direct effect of X and the direct interaction between X and A, one must include Q as well as a product term between Q and A in addition to that between X and A – or, alternatively, stratify not only on X but also on Q.

## IDAGs and generalizability

We now consider the situation where an investigator is not interested in examining interaction per se, but instead in determining an overall effect, such as an average causal effect. If interactions are nevertheless present, sample selection will often cause problems of generalizability, as the average causal effect in the selected sample may differ from that in the target population. In general, this problem will arise if selection depends on variables that influence the causal effect under study.

Standard DAGs can be used to show how sample selection potentially undermines the generalizability of estimates.^20^ For instance Hernan,^21^ and also Westreich *et al*.,^22^ considered a scenario where censoring depended on an unobserved variable that influenced the outcome, and provided DAGs with a selection node for illustration. These standard DAGs are informative about biases that could arise due to non-random sampling, regardless of the chosen effect measure. However, they are not informative about whether, for a chosen effect measure, there actually are interactions with respect to the variables that selection depends on, and thus whether generalizability is in fact compromised.

**Figure 4:**
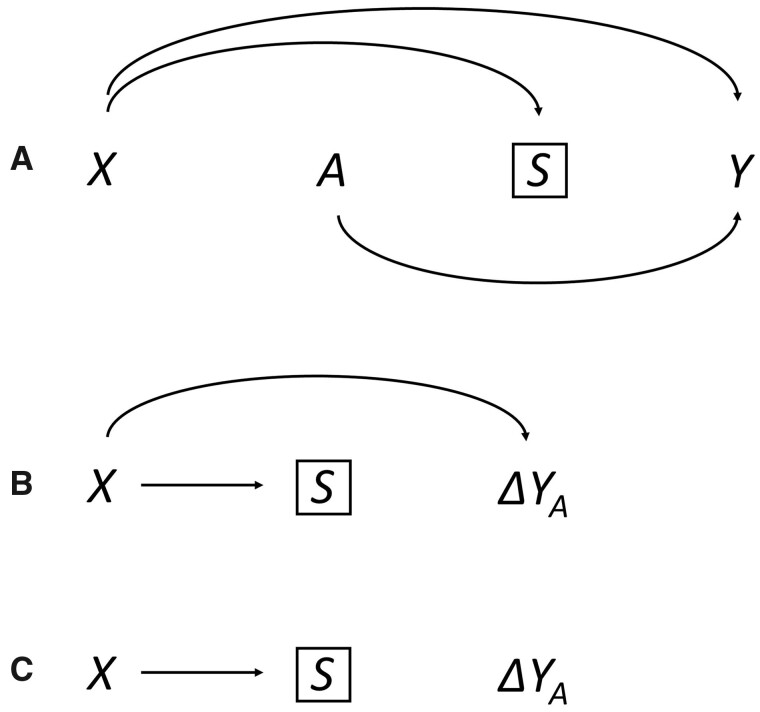
Sample selection potentially compromising generalizability. Individuals are selected based on *S*. *X* may represent socioeconomic status, *A* some treatment, and *Y* a disease. The standard directed acyclic graph (DAG) in panel A is compatible either with the interaction DAG (IDAG) in panel B or the one in panel C, where generalizability is only compromised in the scenario depicted in panel B

In Figure 4, we reproduce the DAGs from Hernan^21^ and from Westreich *et al*.^22^ and display two alternative IDAGs. The treatment of interest is given by A. The first IDAG, shown in Figure 4B, makes it clear that selection on S would compromise generalizability, a conclusion that follows since S and ${\Delta Y}_{A}$ are not d-separated. Selected individuals would tend to have different values on X compared with non-selected individuals, and thus have different causal effects ${\Delta Y}_{A}$. In contrast, this selection issue is not present in Figure 4C. Although S and Y are not d-separated in the DAG, S and ${\Delta Y}_{A}$ are d-separated in the IDAG, as ${\Delta Y}_{A}$ is not influenced by X. The estimate from the study sample would here be valid for the target population.

To restore generalizability, weighting methods are typically applied, where weights are based on the set of variables X which (together with A) block all paths between S and Y.^23–26^ For a given effect measure, this set of variables may however be larger than necessary, as not all of these variables may be related to the effect size. This point has been highlighted by a few recent studies,^27^^,^^28^ but these did not provide a graphical framework for understanding the phenomenon. With our presentation, Figure 4B makes it clear that weighting needs to be done with respect to X, whereas in the scenario displayed in Figure 4C, no weighting is necessary.

As noted, any path involving A is considered ‘automatically’ blocked^20^ in the generalizability framework. For instance, a path S←A→Y would not compromise validity. Conveniently, in the IDAG A is not included and this issue becomes irrelevant.

## Discussion

Standard DAGs are highly informative but lack the ability to depict whether interactions are present on the scale of interest. As a result, their usefulness is limited in terms of understanding the reasons why causal effects vary across individuals, and which interactions to account for. This article introduced a new version of DAGs, the IDAG, to be used for these purposes. Conclusions from the IDAG can be used to achieve internal validity in the sense of unconfounded interaction estimates, and also external validity in the sense of generalizability of estimates of overall effects.

Our framework is distinct from previous attempts to incorporate interactions into DAGs. For example, Weinberg^8^ proposed illustrating interactions by letting arrows point to other arrows or merging arrows in the standard DAG. Although intuitive, this approach is not theoretically consistent with DAG theory. Another previous approach^29^ only applies to synergistic interaction (‘mechanistic’ interaction based on sufficient causes) and yet another one^11^ relies on a mediator between treatment and outcome.

Whereas standard DAGs are nonparametric, we note that the IDAG is parametric in the sense that the absence of an interaction corresponds to a choice of functional form. This makes the IDAG somewhat less general than the standard DAG. However, a functional form is inevitably imposed when conducting (parametric) estimation, and we believe it is rather an advantage that the IDAG narrows the gap between theory and estimation. As for any DAG, assumptions on how the variables in the IDAG are related must be made based on previous evidence.

Several simplifying assumptions were made in this article, in particular that there were no interactions not involving the A variable, and that interactions were constant across individuals. It will be an interesting avenue for future work to elaborate on more general scenarios, where these assumptions are not fulfilled.

## Conclusion

DAGs are useful tools in epidemiology, but one feature of causal relationships which has not been incorporated into the standard framework is interaction. However, interactions can be viewed as ‘effects on effects’ and are therefore conveniently depicted by the IDAG. We expect that our framework will be useful to guide conversations about interaction analyses and to understand whether estimated interactions have a causal interpretation. Describing and guiding analyses in scenarios where sample selection causes lack of generalizability is another benefit.

## Supplementary data

Supplementary data are available at *IJE* online.

## Funding

This work was supported by Forskningsrådet för hälsa, arbetsliv och välfärd (FORTE) [grant number 2017–00414 to U.S.] and Vetenskapsrådet (VR) [grant number 2019–00198 to J.B.].

## Author contributions

A.N. conceived the initial concept and wrote the manuscript. J.B. conceived the idea of using the framework to illustrate generalizability. C.B. contributed with theoretical insights. C.B., U.S. and J.B. contributed to the phrasing of the manuscript.

## Conflict of interest

None declared.

## Supplementary Material

dyaa211_Supplementary_Data
