# Supplementary material for: A directed acyclic graph for interactions
Source: Int J Epidemiol. 2020 Nov 22;50(2):613–9. doi: 10.1093/ije/dyaa211 (PMC8128466; doi:10.1093/ije/dyaa211)
Supplement: dyaa211_Supplementary_Data [file dyaa211_supplementary_data.docx]

**SUPPLEMENTARY APPENDIX**

**Model**

In the Rubin causal model (1–3), each individual is postulated to have two or more potential outcomes, out of which at most one is observed. With two binary treatments of interest ($A$ and $Q$), one may consider four different potential outcomes: $Y^{a=1,q=1}$, $Y^{a=1,q=0}$, $Y^{a=0,q=1}$, and $Y^{a=0,q=0}$, each corresponding to a treatment combination. We invoke the assumption of *causal consistency*, which means that the observed outcome is precisely equal to the potential outcome that corresponds to the actual treatment status.

Assume that a structural causal equation for an outcome is given by the linear equation (1) below, which includes pairwise interactions with $A$. We are interested in the interaction $\gamma$ between $A$ and $Q$. In addition, $A$ interacts with some variable (vector) $X_{1}$. There is also a variable (vector) $X_{2}$, which influences the outcome but does not interact with $A$. To simplify the notation, we here assume that $X_{1}$ and $X_{2}$ are unidimensional. (Also for simplicity, equation (1) does not include any exogenous “noise” or “background” variables, although in general some components of $X_{1}$ or $X_{2}$ could be thought of as exogenous background variables.)

1. $Y=\alpha+\beta_{1}A+\beta_{2}Q+\delta_{1}X_{1}+\delta_{2}X_{2}+\gamma QA+\theta X_{1}A$

The right-hand side variables of equation (1) may be related to each other in a variety of ways, say $A=f_{A}(Q, X_{1},X_{2},\varepsilon_{A})$, $Q=f_{Q}(X_{1},X_{2},\varepsilon_{Q})$, $X_{1}=f_{X_{1}}\left( X_{2},\varepsilon_{X_{1}} \right)$, and $X_{2}=f_{X_{2}}\left( \varepsilon_{X_{2}} \right)$, where the $\varepsilon$’s are exogenous noise variables. Note that we here assume that $X_{1}$ and $X_{2}$ do not depend on $A$ and $Q$.

Based on the model in equation (1), potential outcomes for different combinations of $A$ and $Q$ can be defined by substituting 0 or 1 for $A$ and $Q$:

1. $\left\{ \begin{aligned} \begin{matrix} Y^{a=0,q=0}\left( X_{1},X_{2} \right)=\alpha+\delta_{1}X_{1}+\delta_{2}X_{2} \\ Y^{a=1,q=0}\left( X_{1},X_{2} \right)=\alpha+\beta_{1}+\delta_{1}X_{1}+\delta_{2}X_{2}+\theta X_{1} \end{matrix} \\ \begin{matrix} Y^{a=0,q=1}\left( X_{1},X_{2} \right)=\alpha+\beta_{2}+\delta_{1}X_{1}+\delta_{2}X_{2} \\ Y^{a=1,q=1}\left( X_{1},X_{2} \right)=\alpha+\beta_{1}+\beta_{2}+\delta_{1}X_{1}+\delta_{2}X_{2}+\gamma+\theta X_{1} \end{matrix} \end{aligned} \right.$

Indeed, the potential outcomes can be seen as functions of the $X$ variables; ultimately, they are functions of the exogenous variables $\varepsilon$, as $X$ are functions of $\varepsilon$.

The causal effect of $A$ on $Y$ can be defined based on the expressions for the potential outcomes. For $Q=0$, it is equal to:

1. ${\Delta Y}_{A}\left( 0,X_{1},X_{2} \right)=Y^{a=1,q=0}\left( X_{1},X_{2} \right)-Y^{a=0,q=0}\left( X_{1},X_{2} \right)=\left( \alpha+\beta_{1}+\delta_{1}X_{1}+\delta_{2}X_{2}+\theta X_{1} \right)-\left( \alpha+\delta_{1}X_{1}+\delta_{2}X_{2} \right)=\beta_{1}+\theta X_{1},$

whereas for $Q=1$, it equals:

1. ${\Delta Y}_{A}\left( 1,X_{1},X_{2} \right)=Y^{a=1,q=1}\left( X_{1},X_{2} \right)-Y^{a=0,q=1}\left( X_{1},X_{2} \right)=\left( \alpha+\beta_{1}+\beta_{2}+\delta_{1}X_{1}+\delta_{2}X_{2}+\gamma+\theta X_{1} \right)-\left( \alpha+\beta_{2}+\delta_{1}X_{1}+\delta_{2}X_{2} \right)=\beta_{1}+\gamma+\theta X_{1}$.

Indeed, taking the difference between equations (4) and (3) yields the interaction $\gamma$.

The effect measure ${\Delta Y}_{A}$ is determined by $X_{1}$ and $Q$. Most conveniently, we can write:

1. ${\Delta Y}_{A}=\beta_{1}+\gamma Q+\theta X_{1}.$

Hence, note that an interaction is represented as a *causal effect on an effect measure*.

As ${\Delta Y}_{A}$ is a variable that is defined with a structural causal equation, it can – like any such variable – be represented in a causal graph (4). We refer to a causal graph including this particular variable as an *Interaction DAG* (IDAG). Previous literature has elaborated on several types of causal graphs that include potential outcomes. These include twin networks graphs (5,6), parallel worlds graphs (7,8), and single-world intervention graphs (9,10). However, these have not included nodes contrasting two potential outcomes, as in our setup.

Given that the above $\gamma$ and $\theta$ are different from zero, $Q$ and $X_{1}$ will have arrows pointing to ${\Delta Y}_{A}$ in the IDAG. Typically, the arrows pointing to ${\Delta Y}_{A}$ in the IDAG will be a subset of those pointing to $Y$ in the standard DAG, as there may be $X_{2}$ variables whose nodes have arrows that point to $Y$ but not to ${\Delta Y}_{A}$. Figure A1 shows an example of a possible standard DAG and IDAG (to limit the number of arrows in the standard DAG, the figure assumes that $A$ is not influenced by any variables):

**Figure A1.**


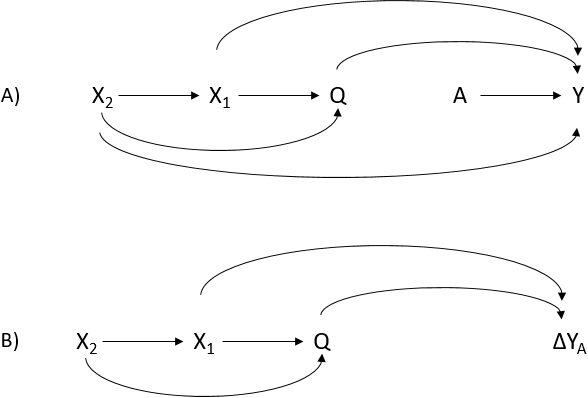


A simple example of a standard DAG (Figure A1A) and an Interaction DAG (IDAG) (Figure A1B).

Apart from shifting the focus in terms of the outcome variable, the IDAG is not much different from the standard DAG, as the relationships between variables do not depend on the graph considered (however, we exclude $A$ from the IDAG as it is not relevant here). Note that, since the IDAG is a causal graph just like a standard DAG, the rules for d-separation (11) apply, although the set of nodes that are d-separated from ${\Delta Y}_{A}$ may be larger than the set of nodes that are d-separated from $Y$. In our example, $X_{2}$ and $Y$ are d-connected, whereas $X_{2}$ and ${\Delta Y}_{A}$ are d-separated, conditional on $Q$ and $X_{1}$. In general, the backdoor paths between $Q$ and ${\Delta Y}_{A}$ may only be a subset of the backdoor paths between $Q$ and $Y$.

**Estimation**

The interactions between $X_{1}$ and $A$ will potentially need to be accounted for in order to unbiasedly estimate $\gamma$, the interaction between $Q$ and $A$. This could be done, for example, by stratifying on $X_{1}$ in addition to on $Q$, or by estimating equation (1) with an Ordinary Least Squares (OLS) regression including the product term $X_{1}A$.^[[1]](#footnote-2)^

Suppose that we examine interaction through the stratification strategy. Below, equation system (6) shows equation (1) stratified on $Q$, and in equation system (7) we have further stratified on $X_{1}$ (we assume that this variable is also binary):

1. $\left\{ \begin{matrix} Y\left( Q=0 \right)=\alpha+\beta_{1}A+\delta_{1}X_{1}+\delta_{2}X_{2}+\theta X_{1}A \\ Y\left( Q=1 \right)=\alpha+\beta_{1}A+\beta_{2}+\delta_{1}X_{1}+\delta_{2}X_{2}+\gamma A+\theta X_{1}A, \end{matrix} \right.$

1. $\left\{ \begin{matrix} \begin{matrix} Y\left( Q=0,X_{1}=0 \right)=\alpha+\beta_{1}A+\delta_{2}X_{2} \\ Y\left( Q=1,X_{1}=0 \right)=\alpha+\beta_{1}A+\beta_{2}+\delta_{2}X_{2}+\gamma A \end{matrix} \\ \begin{matrix} Y\left( Q=0,X_{1}=1 \right)=\alpha+\beta_{1}A+\delta_{1}+\delta_{2}X_{2}+\theta A \\ Y\left( Q=1,X_{1}=1 \right)=\alpha+\beta_{1}A+\beta_{2}+\delta_{1}+\delta_{2}X_{2}+\gamma A+\theta A. \end{matrix} \end{matrix} \right.$

First, assume that $Q$ and $X_{1}$ are (conditionally) independent. This occurs if there is no arrow from $X_{1}$ to $Q$ in the IDAG (and DAG) – or, more generally, if $X_{1}$ and $Q$ are d-separated. A simple example of an IDAG showing such a scenario with independence between $Q$ and $X_{1}$ is displayed in Figure A2A. An average causal effect – “average treatment effect” (ATE) of $A$ estimated from the first stratum in (6) is:

1. $\beta_{1}+\theta\Pr\left[ X_{1}=1|Q=0 \right]=\beta_{1}+\theta\Pr\left[ X_{1}=1 \right]$

whereas an ATE of $A$ estimated from the second stratum in (6) is:

1. $\beta_{1}+\gamma+\theta\Pr\left[ X_{1}=1|Q=1 \right]=\beta_{1}+\gamma+\theta\Pr\left[ X_{1}=1 \right]$

Here, the conditionings on $Q$ were removed, thanks to the assumption that $Q$ and $X_{1}$ are independent.^[[2]](#footnote-3)^ Taking the difference between (9) and (8) yields the interaction $\gamma$, as desired. Note that, to avoid biases, the effects of $X_{1}$ and $X_{2}$ may need to be accounted for when carrying out the estimations, but there is no need to stratify on $X_{1}$; at least not to the extent that the empirical method employed approximately estimates ATEs.

If $Q$ and $X_{1}$ are not (conditionally) independent, the conditionings on $Q$ in (8) and (9) cannot be removed. Figure A2B provides an example of an IDAG showing this case. It is thus not possible to estimate the two equations in (6) and take their difference in order to estimate the interaction; doing so would result in “confounded interaction” (in other words, we would only estimate *effect measure modification*). Instead, one must further stratify on $X_{1}$, as in (7). Estimating the ATE in the first equation of (7) yields $\beta_{1}$ and estimating it in the second yields $\beta_{1}+\gamma$; taking the difference between the second and the first yields the interaction $\gamma$. Similarly, estimating the ATE in the third equation of (7) yields $\beta_{1}+\theta$ and estimating it in the fourth yields $\beta_{1}+\gamma+\theta$; taking the difference between the fourth and the third again yields the interaction $\gamma$. Indeed, the conclusions are in line with the graphs in Figure A2A and A2B: in Figure A2A, there is no need to account for an interaction between $X_{1}$ and $A$ since there is no unblocked backdoor path between $Q$ and ${\Delta Y}_{A}$ through $X_{1}$; in Figure A2B, however, we do need to account for the interaction between $X_{1}$ and $A$ since such an unblocked backdoor path exists.

Moreover, interactions between $X_{2}$ and $A$ do not need to be accounted for, given that interactions between $X_{1}$ and $A$ have been accounted for. This is reflected by the fact that $X_{2}$ does not influence ${\Delta Y}_{A}$, so there is no unblocked backdoor path between $Q$ and ${\Delta Y}_{A}$ through $X_{2}$ when conditioning on $X_{1}$ (i.e. stratifying on $X_{1}$). See Figure A2C for an example.

**Figure A2.**


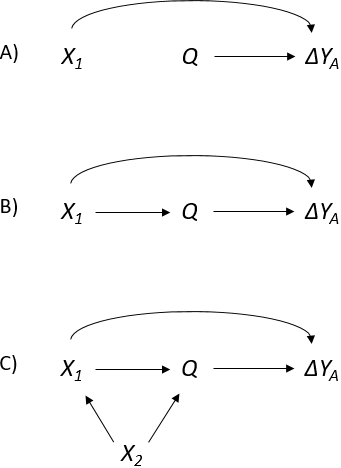


Simple examples of Interaction DAGs (IDAGs) – in Figure A2A, $X_{1}$ and $Q$ are d-separated and, hence, there is no backdoor path between $Q$ and ${\Delta Y}_{A}$ through $X_{1}$ (no need to account for the interaction between $X_{1}$ and $A$), whereas in Figure A2B, $X_{1}$ and $Q$ are d-connected and, hence, there is a backdoor path between $Q$ and ${\Delta Y}_{A}$ through $X_{1}$ (the interaction between $X_{1}$ and $A$ needs to be accounted for). Figure A2C additionally includes a variable $X_{2}$ that is d-separated from ${\Delta Y}_{A}$ conditionally on the included variables and, hence, there is no unblocked backdoor path between $Q$ and ${\Delta Y}_{A}$ through $X_{2}$ (an interaction between $X_{2}$ and $A$ does not need to be accounted for, given that we have accounted for the interaction between $X_{1}$ and $A$).

Note that, strictly speaking, ATEs (which played a role in the above discussions) are not estimated by standard regression methods such as OLS. Instead, an OLS regression estimates a *weighted* average of causal effects, where the weights are proportional to the conditional variance of treatment (12,13). In the scenario where $Q$ and $X_{1}$ are conditionally independent (and assuming that $A$ is not randomized), OLS regressions applied to the two strata in equation system (6), not accounting for the product terms $X_{1}A$, may weigh the causal effects of $A$ in different ways. As a result, an accurate estimate of the interaction $\gamma$ may not be obtained by taking the difference between the two strata-specific estimates. Weighted regression methods that estimate ATEs may thus need to be used, unless *all* interactions involving variables that are confounders (in the standard DAG) are being accounted for.

If outcomes are binary, one can envision a model similar to equation (1), but where the $Y$ on the left-hand side has been replaced by a probability of $Y$ being positive, or some function of this probability. Different functions correspond to different scales, e.g. with a logarithmic function, $\gamma\neq0$ represents that an interaction is present on a relative risk scale, whereas, if the left-hand side is simply the untransformed probability of $Y$ being positive, $\gamma\neq0$ represents that an interaction is present on an absolute risk scale. Our framework can be used to display interactions in these cases as well. In terms of estimation, the qualification in the last paragraph still applies, i.e. that standard regression models for binary outcomes do not quite estimate average effects, with similar implications as above.

Our framework does not hinge on the direction of causality between the variables influencing the outcome $Y$. For example, while we assumed above that $X_{1}$ and $X_{2}$ did not depend on $A$ or $Q$, the opposite scenario, where $X_{1}$ or $X_{2}$ might be mediators of the relationship between $A$ or $Q$ and $Y$ is conceivable as well. The interaction $\gamma$, identified as discussed above, would then only represent a *direct* interaction. To measure *total* interaction, mediators should generally be omitted the analysis.

**Examples with an unobserved confounder**

Finally, we discuss a couple of scenarios with an unobserved confounder $U$. Assume that the outcome is determined according to equation (10):

1. $Y=\alpha+\beta_{1}A+\beta_{2}Q+\delta_{1}X+\delta_{2}U+\gamma QA+\theta UA$.

As before, we assume that all right-hand side variables are binary.

A standard DAG is given in Figure A3A and two alternative IDAGs in Figures A3B and A3C.

We consider two scenarios:

1. There is a direct interaction between $U$ and $A$ ($\theta\neq0$; Figure A3B) and
2. There is no direct interaction between $U$ and $A$ ($\theta=0$; Figure A3C).

**Figure A3.**


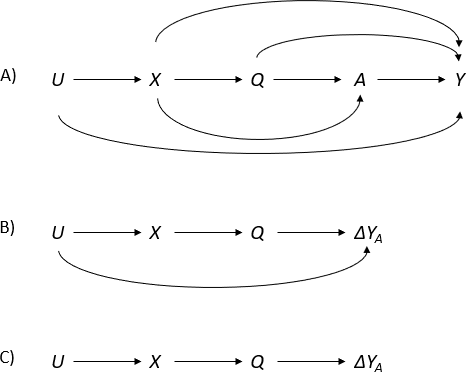


An example of a standard Directed Acyclic Graph (DAG) and two alternative Interaction DAGs (IDAGs) with an unobserved confounder.

We first consider scenario 1 (Figure A3B). Suppose that interaction is examined through stratification on $Q$. Equation (10) yields:

1. $\left\{ \begin{matrix} Y\left( Q=0 \right)=\alpha+\beta_{1}A+\delta_{1}X+\delta_{2}U+\theta UA \\ Y\left( Q=1 \right)=\alpha+\beta_{1}A+\beta_{2}+\delta_{1}X+\delta_{2}U+\gamma A+\theta UA. \end{matrix} \right.$

Causal effects vary according to the value of $U$. Since, according to the DAGs, $U$ is related to $X$ and $X$ is related to $Q$, $U$ is in turn also related to $Q$, and estimates in different $Q$-strata would tend to reflect individuals with different values of $U$. Comparisons of average effects of $A$ across $Q$-strata would therefore not yield conclusions about interaction in a causal sense.

Suppose, however, that we also stratify on $X$:

1. $\left\{ \begin{matrix} \begin{matrix} Y\left( Q=0,X=0 \right)=\alpha+\beta_{1}A+\delta_{2}U+\theta UA \\ Y\left( Q=1,X=0 \right)=\alpha+\beta_{1}A+\beta_{2}+\delta_{2}U+\gamma A+\theta UA \end{matrix} \\ \begin{matrix} Y\left( Q=0,X=1 \right)=\alpha+\beta_{1}A+\delta_{1}+\delta_{2}U+\theta UA \\ Y\left( Q=1,X=1 \right)=\alpha+\beta_{1}A+\beta_{2}+\delta_{1}+\delta_{2}U+\gamma A+\theta UA. \end{matrix} \end{matrix} \right.$

As we have conditioned on $X$, the distribution of $U$ is independent of $Q$ ($U$ and $Q$ are d-separated in Figures A3, conditional on $X$), so ATE estimates across different $Q$-strata but within the same $X$-stratum will reflect individuals that are similar with respect to $U$. Hence, by comparing treatment effect estimates across strata where $X$ is fixed but $Q$ differs, the interaction effect $\gamma$ may be determined. Note that the conclusions are in line with the IDAG in Figure A3B, showing that the backdoor path between $Q$ and ${\Delta Y}_{A}$ needs to be blocked by the variable $X$.

In scenario 2, illustrated in Figure A3C, stratification on $Q$ simply gives:

1. $\left\{ \begin{matrix} Y\left( Q=0 \right)=\alpha+\beta_{1}A+\delta_{1}X+\delta_{2}U \\ Y\left( Q=1 \right)=\alpha+\beta_{1}A+\beta_{2}+\delta_{1}X+\delta_{2}U+\gamma A. \end{matrix} \right.$

$U$ is the only unobserved variable and there is no effect heterogeneity conditional on $Q$. Estimated treatment effects are simply $\beta_{1}$ and $\beta_{1}+\gamma$; whose difference yields the interaction $\gamma$. The estimates based on (13) are unconfounded since, as follows from the standard DAG, $A$ is independent of $U$ given the conditionings on $X$ and $Q$. No further stratification or inclusion of product terms is necessary. Again, note that the conclusion is in line with the IDAG.

**References**

1. Rubin D. Causal inference using potential outcomes: design, modeling, decisions. *J Am Stat Assoc* 2005;**100**:322–31.

2. Imbens GW, Rubin DB. Rubin causal model. In: Durlauf SN, Blume LE, editors. The New Palgrave Dictionary of Economics. 2nd ed. New York, NY: Palgrave Macmillan; 2008.

3. Robins JM, Hernan MA, Brumback B. Marginal structural models and causal inference in epidemiology. *Epidemiol* 2000;**11**:550–60.

4. Pearl J. Structural model semantics. In: Causality. 2nd ed. Cambridge, UK: Cambridge University Press; 2009:202–15.

5. Balke A, Pearl J. Counterfactual probabilities: Computational methods, bounds and applications. In: Proceedings of UAI-94. 1994:46–54.

6. Balke A. Probabilistic evaluation of counterfactual queries. In: Pearl, J. 1994:230–7.

7. Avin C, Shpitser I, Pearl J. Identifiability of path-specific effects. In: International Joint Conference on Artificial Intelligence. 2005:357–63.

8. Shpitser I, Pearl J. Complete identification methods for the causal hierarchy. *J Mach Learn Res* 2008;**9**:1941–79.

9. Richarson TS, Robins JM. Single world intervention graphs (SWIGs): A unification of the counterfactual and graphical approaches to causality. 2013. (Center for the Statistics and the Social Sciences, University of Washington Series).

10. Richardson TS, M RJ. Single world intervention graphs: a primer. In: Second UAI workshop on causal structure learning, Bellevue, Washington. 2013.

11. Pearl J. Causal diagrams for empirical research. *Biometrika* 1995;**82**:669–710.

12. Angrist JD, Pischke J-S. Regression meets matching. In: Mostly Harmless Econometrics. Princeton, NJ: Princeton University Press; 2009. p. 69–80.

13. Aronow PM, Samii C. Does Regression Produce Representative Estimates of Causal Effects? *Am J Pol Sci* 2016;**60**:250–67.

14. VanderWeele TJ. On the distinction between interaction and effect modification. *Epidemiology* 2009;**20**:863–71.

15. Hernan MA, Robins JM. IP weighting and marginal structural models. In: Causal inference: What if? Boca Raton, FL: Chapman & Hall/CRC; 2020:149–60.

1. Another option is to estimate an inverse-probability-of-treatment weighted regression (3,14,15), where the weights are constructed based on a full set of confounders. Stratifying on $A$ is yet another option that we disregard here, as we consider $A$ as the treatment of primary interest, a variable that researchers do normally not stratify on. [↑](#footnote-ref-2)
2. More generally, if $Q$ and $X_{1}$ are only *conditionally* independent given $X_{2}$, we will need to stratify on $X_{2}$ in order to be able to remove the conditionings on $Q$ as above. In graphical terms, this is a scenario like in Figure A2A but where there is also an $X_{2}$ variable with arrows to both $Q$ and $X_{1}$.The variable $X_{2}$ here blocks a backdoor path. [↑](#footnote-ref-3)
